# Case Report: “We are not alone”: shared vulnerability and peer solidarity as catalysts for healing in child sexual abuse

**DOI:** 10.3389/fpsyg.2026.1749284

**Published:** 2026-02-16

**Authors:** S. V. Silvia, J. Balamurugan

**Affiliations:** Department of Social Sciences, School of Social Sciences and Languages, Vellore Institute of Technology, Vellore, India

**Keywords:** adolescent girls, case study report, child sexual abuse, psychosocial support, rehabilitation

## Abstract

**Background:**

Child sexual abuse (CSA) profoundly affects children's emotional and developmental well-being. For many survivors, the inability to fully understand the abuse during childhood is used by perpetrators to maintain control and silence, creating long-lasting psychological consequences.

**Aim:**

This study explores the two adolescent girls make meaning of their CSA experiences and how sharing their narratives with each other contributes to their healing and personal growth.

**Methods:**

This study draws on in-depth interviews with two female adolescent survivors of CSA. A reflexive thematic analysis approach was used to explore how they make sense of their experiences and how shared storytelling shaped their recovery.

**Results:**

The findings highlight the restorative power of peer connection. As the two adolescents exchanged their narratives, they recognized shared forms of vulnerability rooted in similar socioeconomic and family contexts. This mutual recognition fostered emotional safety, validation, and a unique form of solidarity that supported their healing and personal growth.

**Conclusions:**

The study suggests that recovery for adolescent CSA survivors can be meaningfully strengthened through relational spaces where survivors share their stories with peers who understand similar forms of pain. Such shared narrative environments can cultivate compassion, relational closeness, and emerging post-traumatic growth.

## 1 Introduction

Child sexual abuse (CSA) is recognized globally as a profound violation of human rights and a critical public health issue, with devastating consequences for the survivor's psychological, physical, and social well-being (World Health Organization, 2022). The trauma inflicted by CSA is distinct in its etiology and impact, largely stemming from the child's developmental inability to cognitively and emotionally process the exploitation. This inherent vulnerability is often systematically weaponized by the perpetrator, who utilizes secrecy, manipulation, and coercion to ensure compliance and silence, thereby embedding deep-seated feelings of shame, guilt, and isolation in the victim (Collin-Vézina et al., 2013).

For adolescent survivors, the path to rehabilitation is exceptionally complex. They must navigate the normative developmental challenges of identity formation, autonomy, and intimacy while simultaneously contending with the aftermath of profound betrayal trauma (Foster et al., 2019; Manukrishnan and Bhagabati, 2023). Traditional models of psychosocial support, while foundational, often operate from an individual-centric framework. These models can sometimes fall short in fully addressing the pervasive sense of alienation and the shattered belief in a safe and predictable world that CSA survivors experience (Knight, 2015). The very nature of the trauma—a betrayal of trust by someone who often holds a position of power or care—creates a relational wound that, research suggests, requires a relational comfort (Spiegel, 1998). Consequently, there is a growing consensus that effective rehabilitation must extend beyond symptom reduction to foster reconnection, safety, and empowerment within a therapeutic community.

Within this context, the role of peer support and shared experience emerges as a potentially powerful, yet under-explored, mechanism of healing. The therapeutic factor of “universalization,” as outlined in group therapy principles, allows individuals to recognize that their suffering is not unique, thereby mitigating feelings of isolation (Parcover et al., 2006). However, when this shared experience is rooted not only in the commonality of abuse but also in parallel socioeconomic and familial vulnerabilities, it may forge a particularly potent form of solidarity. This shared vulnerability can become the foundation for a unique therapeutic intimacy—a mutual compassion that validates experiences and challenges the perpetrator's-imposed narrative of shame and solitude.

While the literature establishes the efficacy of group therapy in general, there is a need for deeper qualitative inquiry into the specific processes through which peer solidarity, born from shared life contexts, functions as an active catalyst for healing in CSA rehabilitation. This study seeks to address this gap by presenting a detailed analysis of two case studies of adolescent girls navigating recovery from CSA. It explores their journeys through a psychosocial support program, examining the complexities of their pain and the significant role that mutual understanding and peer solidarity play in facilitating a pathway toward healing.

## 2 Methodology

### Aim and research question

To address the central research question “How do adolescent girls recover from the experience of child sexual abuse (CSA)?”, a qualitative case study methodology was employed. Data were gathered through semi-structured interviews, which allowed for the collection of rich, narrative data while providing the flexibility to explore emergent themes sensitively. The interview protocol was carefully constructed to investigate key domains of the recovery process, including the experience of psychosocial support, the emotional landscape of pain and healing, the role of interpersonal relationships, and the influence of shared socioeconomic and familial vulnerabilities. This methodological approach was chosen to center the voices and lived experiences of the survivors, thereby illuminating the nuanced pathways of their rehabilitation.

### Philosophical and theoretical influences

Employing an interpretivist/constructivist paradigm with a case study approach, this study reports on two individual's experiences of CSA as the construction of subjective reality. Thus, an in-depth interview served as a primary source of providing phenomenal perspectives to explore the key objective (Creswell, 1994).

### Case study design

A case study focuses on particularization, focusing on the uniqueness of a case rather than its differences from others, according to Mills et al. (2010). The choice of a case study design is not to enhance the production of generalizations. Cases are of interest for their uniqueness and commonality. Another way to classify a case study is as an intrinsic case study whose purpose is first and foremost to better understand this case (Denzin and Lincoln, 2011).

### Use of reflexive thematic analysis

Reflexive Thematic Analysis (RTA) ontological and epistemological stance should be made clear, according to Braun and Clarke (2019). Perception, context, and culture all have an impact on how reality is perceived, with a focus on experience that is situated within a larger social context (Braun et al., 2019). A qualitative analysis technique called Reflexive Thematic Analysis (RTA) emphasizes the interpretation and comprehension of participants' subjective experiences (Drinkwater et al., 2022).

### Rigor

The current study used a 15-item checklist, as suggested by Braun and Clarke (2013), to guarantee the quality of research and findings and to generate high-quality RTA. Every aspect of research methods, including transcription, coding, analysis, and writing up, is covered by the checklist.

### Context

The study was conducted in collaboration with XXXXX (Non-Governmental Organization—NGO) operating in a district of Tamil Nadu, India. This NGO partners with a local school to implement psychosocial support programs, with a specific focus on the welfare and development of girl children. Through its ongoing engagement, the NGO identified several students as survivors of child sexual abuse. Recognizing the need for specialized psychological intervention and the value of documenting their recovery processes, the NGO referred these cases to the researcher for the present study.

The researcher, operating in a dual role as a psychologist and investigator, conducted the research with formal ethical approval from the institution board obtained in accordance with the Indian Council of Medical Research (ICMR) guidelines. The NGO-appointed psychologist has provided psychological support to the participants. Selected participants have already received psychological support by being made aware of their circumstances. The participants received psychological first aid during the interview process, including the introduction of the terms “pause,” “stop,” and “continue.” They have utilized it. The researcher's laptop was equipped with a protection key to safeguard the confidentiality of the interview recordings, which were recorded using an audio recorder.

### Ethics

The research is a part of the doctoral thesis and has been formally approved by the Institutional Ethics Committee [VIT/IECH/2024/15 IECH/20 April 2024/13]. Since it is a study involving children, the forms of informed consent and assent of different kinds (for children and their parents/guardian) have been prepared and approved by the Institutional Ethics Committee.

The parents/guardian and participants provided informed consent to participate in the data collection and allowed for these data to be published in the present paper.

To preserve emotional integrity, participant-led interviews included grounding techniques, ongoing distress monitoring, and post-interview decompression. Confidentiality was ensured through pseudonyms, the blinding of identifying details, secure data handling, and member checking—returning control to participants over their narratives. Support measures included pre-identified local mental health resources, an on-call counselor, and well-being checks. The research team also received mandatory clinical supervision to mitigate vicarious trauma and uphold the ethical principle of non-maleficence.

## 3 Results

### Summary of participant's testimony

#### Case: 1—SA

##### Context of abuse

The participant, a 17-year-old from a disadvantaged background, survived child sexual abuse (CSA) by two perpetrators in tutorial settings. The first abuse occurred in eighth standard by a male tutor, who used “advice” as a pretext to progress from inappropriate touching to severe sexual assault. The second, recent abuse at age 17 was perpetrated by the husband of her female tutor, who employed grooming tactics—escalating from “brotherly” touches to sexual assault and lewd comments.

##### Initial aftermath and disclosure

After the first abuse, the participant's disclosure to her family led to a confrontation where the tutor denied the allegations. Despite relatives urging a formal complaint, her mother withdrew her from the tuition to protect her studies and reputation, avoiding legal action. During the second abuse, the perpetrator's wife (the teacher) witnessed the behavior but dismissed it as “fun,” ignoring the victim's pleas. The participant was further traumatized by peers at the tuition center who publicly mocked and questioned her.

##### Evolving recovery trajectory

The participant exhibits resilience and clear insight. She redirects blame from herself to the perpetrators and enabling societal structures, challenging victim-shaming culture and gendered concepts like “virginity.” She resolves to educate her future children about “good and bad touch” regardless of gender and to act immediately on any disclosure of abuse—contrasting her own experience. While suffering lingering impacts like nightmares and confusion, her testimony concludes with a determined commitment to self-protection and future prevention, emphasizing that the duty of prevention lies not with potential victims but with society.

#### Case: 2—KS

##### Context of abuse

The participant, a 17-year-old from a single-parent household, endured child sexual abuse (CSA) across two periods. The first incident occurred in early childhood (age 6–7) by a 19-year-old neighbor who lured her to his home. Due to her age, she lacked the comprehension to understand the abuse, only recalling physical distress. The second, ongoing abuse is perpetrated by a man in his forties, assisted by his wife. The couple uses grooming and normalization tactics; the wife enables the abuse by dismissing it. The abuse escalated during a terrifying outing where, after emotional pressure, the perpetrator groped her and bit her on the back, leaving her profoundly violated. The childhood abuse is compounded by the perpetrator's recent, taunting reminders.

##### Initial aftermath and disclosure

A central theme is the isolation created by fear and stigma. Despite having a supportive mother, the participant actively withholds disclosure to protect her. She fears the abuse would be used as a weapon against her, that she would be mocked, and her character questioned. Crucially, she fears revealing the truth would be an unbearable burden for her single mother, potentially causing “chaos,” a responsibility she feels she must prevent.

##### Evolving recovery trajectory

The participant critically analyzes the abuse dynamics, noting perpetrators strategically target vulnerability, as she was seen as less likely to complain. She identifies that consequences like pregnancy and social shame fall disproportionately on girls, which protects male perpetrators. She concludes the root cause is the “mindset of men” and their entitlement. Her proposed solutions focus on prevention: parents must teach “good and bad touch” before puberty, as her own late education left her defenseless. Ultimately, the testimony reveals lingering trauma, with intrusive memories and fear upon encountering her perpetrators. She carries the silent burden of abuse to protect her loved ones from the truth that haunts her.

##### Peer interactions from two cases

The participant found strength and validation through peer solidarity. She discovered that at least three other girls from the same tuition were facing similar abuse from the same man. They confided in each other, creating a private support system and using code language to communicate. This shared experience confirmed that the abuse was real and not their fault. However, this solidarity was tempered by fear. They were acutely aware of the social repercussions, having seen another victim publicly blamed and her “name spoiled” when she spoke out. The participant's own mother, while believing her, chose to withdraw her under a false pretext to avoid a public confrontation, explicitly stating her fear of the consequences of fighting for justice. The participant provides a sharp analysis of the perpetrators' motives and methods. She observes that the first perpetrator selectively targeted girls from poorer families, calculating they would be less likely to report him. The second perpetrator, she notes, targeted girls based on their physical appearance (preferring those who were “slim”) and used psychological manipulation, alternating between threats about her academic future and philosophical justifications about “enjoying life” through engaging girls in the inappropriate acts.

## 4 Discussion

### Interpreting the cases: shared vulnerability as a foundation for peer-supported rehabilitation

The findings from this analysis argue for a critical expansion of therapeutic frameworks for adolescent CSA survivors, moving beyond an exclusively individual, clinical model to formally incorporate the healing potential of peer solidarity grounded in shared socioeconomic and familial vulnerability. Children with experiences of Child sexual abuse will have trust issues in reporting the conflict and it continues to happen as they grow up. (Alyce et al., 2024; Draucker et al., 2011). Conventional approaches rightly address individual trauma, yet they often overlook how organic connections among survivors from similar marginalized backgrounds can serve as a powerful, unanticipated catalyst for rehabilitation.

One participant's ability to process her abuse was linked to confiding in a peer, forcing the research team to balance confidentiality with the therapeutic benefit of this mutual aid. The second participant's isolation was rooted in a desire to protect her financially strained, single mother, fearing that seeking justice would further victimize her family (Lashkay et al., 2023). Though, all other members in the tuition were pointing out the girl who's also undergone abuse apart from the participants employed in the present study. That girl has been mocked and disrespected for raising her Child sexual abuse allegations against the tutor. In one study, data show that group loyalties provide a psychological motivation to disbelieve child abuse allegations (Minto et al., 2016).

In the cases examined, individual healing was profoundly facilitated by peer relationships that provided a sanctuary from familial pressure and a space for stories to be heard without judgment, directly countering internalized shame and isolation. This suggests that financial precarity and fractured family systems, typically viewed as risk factors, can also become conduits for mutual recognition and a collectivist model of recovery, particularly for adolescents who may distrust institutional support and rely primarily on peers for validation (Bowker and Weingarten, 2022; Costello et al., 2024).

The mechanism of this healing is rooted in the dynamics of shared vulnerability and relational intimacy. Through reciprocal self-disclosure, peers normalize traumatic experiences, reduce feelings of otherness, and build trust—a process aligned with Reis and Shaver's intimacy model and Relational Cultural Theory, which posits that resilience is cultivated through growth-fostering relationships (Miller, 1997; Mereish and Poteat, 2015) rather than individual traits alone. However, these organic bonds are fragile and carry risks, including confidentiality breaches, emotional burden, and the potential for co-rumination, where shared vulnerability reinforces distress rather than alleviating it. Furthermore, as illustrated by the peer rejection one survivor faced, social identity dynamics can lead groups to disbelieve or stigmatize allegations, isolating the victim further. This underscores the need for professional facilitation to harness the therapeutic potential of peer solidarity while ensuring emotional safety (Birrell et al., 2025; Muldoon et al., 2024).

Clinically, this implies a proactive shift in practice. Interventions should include systematic screening for isolation and shared social contexts during intake, followed by the offer of professionally moderated peer support groups composed of individuals with aligned backgrounds. The clinician's role evolves to include creating “containment”—a balanced space that avoids both emotional avoidance and retraumatizing flooding—while guiding reciprocal vulnerability toward growth. Ethically, this model requires navigating confidentiality carefully and structuring interactions to minimize relational risks.

Future research should empirically validate this approach across broader and more diverse populations, examine long-term recovery outcomes, and refine protocols for integrating peer-based support into trauma-informed care. Ultimately, this discussion posits that healing from CSA is not merely an individual journey but a relational process. By reframing shared social vulnerability not just as a risk marker but as a foundation for therapeutic connection, clinical practice can better address the complex social ecology of trauma and foster resilience through collective, as well as personal, strength (Tolendi, 2024; Narang et al., 2019; Blikhar, 2024).

## 5 Conclusion

Like any therapeutic intervention, rehabilitation from child sexual abuse occurs within a complex social ecosystem. For adolescents from marginalized or financially precarious backgrounds, the journey of healing is often inextricably linked to their social and familial vulnerabilities. While traditional clinical models rightly focus on the individual's intrapsychic trauma, our findings indicate that for these populations, the isolation imposed by these very vulnerabilities can be a significant barrier to recovery. The peer solidarity that emerged between participants—forged in the common ground of their socioeconomic realities—proved to be a powerful, unanticipated catalyst for healing. It mitigated shame, validated their experiences, and fostered a collective resilience. Therefore, it may be imperative for rehabilitation frameworks to proactively create safe, professionally facilitated spaces where such therapeutic peer connections, built on shared vulnerability, can form and flourish.

## 6 Clinical and practical implications

Those designing and implementing psychosocial support programs for adolescent survivors of child sexual abuse should consider the following:

- **Routinely Assess Social Context:** During intake and assessment, clinicians should actively explore not just the individual trauma narrative, but also the client's perceived social isolation, familial pressures, and socioeconomic stressors.
- **Facilitate Peer Connection:** Where appropriate and with informed consent, practitioners should consider creating structured, professionally moderated peer support groups for survivors from similar backgrounds. This shared context of vulnerability can be a foundational element for building trust and therapeutic intimacy.
- **Reframe Vulnerability as a Potential Strength:** Instead of viewing socioeconomic disadvantage solely as a risk factor, therapeutic models can be adapted to recognize the potential for shared experience to become a source of mutual support, validation, and collective empowerment, thereby accelerating the rehabilitation process.

## Data Availability

The datasets presented in this article are not readily available because of ethical and privacy restrictions. Requests to access the datasets should be directed to the corresponding author.
